# 30-Day Morbidity and Mortality of Bariatric Surgery During the COVID-19 Pandemic: a Multinational Cohort Study of 7704 Patients from 42 Countries

**DOI:** 10.1007/s11695-021-05493-9

**Published:** 2021-07-30

**Authors:** Rishi Singhal, Christian Ludwig, Gavin Rudge, Georgios V. Gkoutos, Abd Tahrani, Kamal Mahawar, on behalf of the GENEVA Collaborators, Michał Pędziwiatr, Piotr Major, Piotr Zarzycki, Athanasios Pantelis, Dimitris P. Lapatsanis, Georgios Stravodimos, Chris Matthys, Marc Focquet, Wouter Vleeschouwers, Antonio G Spaventa, Carlos Zerrweck, Antonio Vitiello, Giovanna Berardi, Mario Musella, Alberto Sanchez-Meza, Felipe J. Cantu, Fernando Mora, Marco A. Cantu, Abhishek Katakwar, D. Nageshwar Reddy, Haitham Elmaleh, Mohammad Hassan, Abdelrahman Elghandour, Mohey Elbanna, Ahmed Osman, Athar Khan, Laurent Layani, Nalini Kiran, Andrey Velikorechin, Maria Solovyeva, Hamid Melali, Shahab Shahabi, Ashish Agrawal, Apoorv Shrivastava, Ankur Sharma, Bhavya Narwaria, Mahendra Narwaria, Asnat Raziel, Nasser Sakran, Sergio Susmallian, Levent Karagöz, Murat Akbaba, Salih Zeki Pişkin, Ahmet Ziya Balta, Zafer Senol, Emilio Manno, Michele Giuseppe Iovino, Ahmed Osman, Mohamed Qassem, Sebastián Arana-Garza, Heitor P. Povoas, Marcos Leão Vilas-Boas, David Naumann, Jonathan Super, Alan Li, Basil J. Ammori, Hany Balamoun, Mohammed Salman, Amrit Manik Nasta, Ramen Goel, Hugo Sánchez-Aguilar, Miguel F. Herrera, Adel Abou-Mrad, Lucie Cloix, Guilherme Silva Mazzini, Leonardo Kristem, Andre Lazaro, Jose Campos, Joaquín Bernardo, Jesús González, Carlos Trindade, Octávio Viveiros, Rui Ribeiro, David Goitein, David Hazzan, Lior Segev, Tamar Beck, Hernán Reyes, Jerónimo Monterrubio, Paulina García, Marine Benois, Radwan Kassir, Alessandro Contine, Moustafa Elshafei, Sueleyman Aktas, Sylvia Weiner, Till Heidsieck, Luis Level, Silvia Pinango, Patricia Martinez Ortega, Rafael Moncada, Victor Valenti, Ivan Vlahović, Zdenko Boras, Arnaud Liagre, Francesco Martini, Gildas Juglard, Manish Motwani, Sukhvinder Singh Saggu, Hazem Al Moman, Luis Adolfo Aceves López, María Angelina Contreras Cortez, Rodrigo Aceves Zavala, Christine D’Haese, Ivo Kempeneers, Jacques Himpens, Andrea Lazzati, Luca Paolino, Sarah Bathaei, Abdulkadir Bedirli, Aydın Yavuz, Çağrı Büyükkasap, Safa Özaydın, Andrzej Kwiatkowski, Katarzyna Bartosiak, Maciej Walędziak, Antonella Santonicola, Luigi Angrisani, Paola Iovino, Rossella Palma, Angelo Iossa, Cristian Eugeniu Boru, Francesco De Angelis, Gianfranco Silecchia, Abdulzahra Hussain, Srivinasan Balchandra, Izaskun Balciscueta Coltell, Javier Lorenzo Pérez, Ashok Bohra, Altaf K Awan, Brijesh Madhok, Paul C Leeder, Sherif Awad, Waleed Al-Khyatt, Ashraf Shoma, Hosam Elghadban, Sameh Ghareeb, Bryan Mathews, Marina Kurian, Andreas Larentzakis, Gavriella Zoi Vrakopoulou, Konstantinos Albanopoulos, Ahemt Bozdag, Azmi Lale, Cuneyt Kirkil, Mursid Dincer, Ahmad Bashir, Ashraf Haddad, Leen Abu Hijleh, Bruno Zilberstein, Danilo Dallago de Marchi, Willy Petrini Souza, Carl Magnus Brodén, Hjörtur Gislason, Kamran Shah, Antonio Ambrosi, Giovanna Pavone, Nicola Tartaglia, S. Lakshmi Kumari Kona, K. Kalyan, Cesar Ernesto Guevara Perez, Miguel Alberto Forero Botero, Adrian Covic, Daniel Timofte, Madalina Maxim, Dashti Faraj, Larissa Tseng, Ronald Liem, Gürdal Ören, Evren Dilektasli, Ilker Yalcin, Hudhaifa AlMukhtar, Mohammed Al Hadad, Rasmi Mohan, Naresh Arora, Digvijaysingh Bedi, Claire Rives-Lange, Jean-Marc Chevallier, Tigran Poghosyan, Hugues Sebbag, Lamia Zinaï, Saadi Khaldi, Charles Mauchien, Davide Mazza, Georgiana Dinescu, Bernardo Rea, Fernando Pérez-Galaz, Luis Zavala, Anais Besa, Anna Curell, Jose M. Balibrea, Carlos Vaz, Luis Galindo, Nelson Silva, José Luis Estrada Caballero, Sergio Ortiz Sebastian, João Caetano Dallegrave Marchesini, Ricardo Arcanjo da Fonseca Pereira, Wagner Herbert Sobottka, Felipe Eduardo Fiolo, Matias Turchi, Antonio Claudio Jamel Coelho, Andre Luis Zacaron, André Barbosa, Reynaldo Quinino, Gabriel Menaldi, Nicolás Paleari, Pedro Martinez-Duartez, Gabriel Martínez de Aragon Ramírez de Esparza, Valentin Sierra Esteban, Antonio Torres, Jose Luis Garcia-Galocha, Miguel Josa, Jose Manuel Pacheco-Garcia, Maria Angeles Mayo-Ossorio, Pradeep Chowbey, Vandana Soni, Hercio Azevedo de Vasconcelos Cunha, Michel Victor Castilho, Rafael Meneguzzi Alves Ferreira, Thiago Alvim Barreiro, Alexandros Charalabopoulos, Elias Sdralis, Spyridon Davakis, Benoit Bomans, Giovanni Dapri, Koenraad Van Belle, Mazen Takieddine, Pol Vaneukem, Esma Seda Akalın Karaca, Fatih Can Karaca, Aziz Sumer, Caghan Peksen, Osman Anil Savas, Elias Chousleb, Fahad Elmokayed, Islam Fakhereldin, Hany Mohamed Aboshanab, Talal Swelium, Ahmad Gudal, Lamees Gamloo, Ayushka Ugale, Surendra Ugale, Clara Boeker, Christian Reetz, Ibrahim Ali Hakami, Julian Mall, Andreas Alexandrou, Efstratia Baili, Zsolt Bodnar, Almantas Maleckas, Rita Gudaityte, Cem Emir Guldogan, Emre Gundogdu, Mehmet Mahir Ozmen, Deepti Thakkar, Nandakishore Dukkipati, Poonam Shashank Shah, Shashank Subhashchandra Shah, Simran Shashank Shah, Md Tanveer Adil, Periyathambi Jambulingam, Ravikrishna Mamidanna, Douglas Whitelaw, Md Tanveer Adil, Vigyan Jain, Deepa Kizhakke Veetil, Randeep Wadhawan, Antonio Torres, Max Torres, Tabata Tinoco, Wouter Leclercq, Marleen Romeijn, Kelly van de Pas, Ali K. Alkhazraji, Safwan A. Taha, Murat Ustun, Taner Yigit, Aatif Inam, Muhammad Burhanulhaq, Abdolreza Pazouki, Foolad Eghbali, Mohammad Kermansaravi, Amir Hosein Davarpanah Jazi, Mohsen Mahmoudieh, Neda Mogharehabed, Gregory Tsiotos, Konstantinos Stamou, Francisco J. Barrera Rodriguez, Marco A. Rojas Navarro, Omar MOhamed Torres, Sergio Lopez Martinez, Elda Rocio Maltos Tamez, Gustavo A. Millan Cornejo, Jose Eduardo Garcia Flores, Diya Aldeen Mohammed, Mohamad Hayssam Elfawal, Asim Shabbir, Kim Guowei, Jimmy By So, Elif Tuğçe Kaplan, Mehmet Kaplan, Tuğba Kaplan, DangTuan Pham, Gurteshwar Rana, Mojdeh Kappus, Riddish Gadani, Manish Kahitan, Koshish Pokharel, Alan Osborne, Dimitri Pournaras, James Hewes, Errichetta Napolitano, Sonja Chiappetta, Vincenzo Bottino, Evelyn Dorado, Axel Schoettler, Daniel Gaertner, Katharina Fedtke, Francisco Aguilar-Espinosa, Saul Aceves-Lozano, Alessandro Balani, Carlo Nagliati, Damiano Pennisi, Andrea Rizzi, Francesco Frattini, Diego Foschi, Laura Benuzzi, Chirag Parikh, Harshil Shah, Enrico Pinotti, Mauro Montuori, Vincenzo Borrelli, Jerome Dargent, Catalin A. Copaescu, Ionut Hutopila, Bogdan Smeu, Bart Witteman, Eric Hazebroek, Laura Deden, Laura Heusschen, Sietske Okkema, Theo Aufenacker, Willem den Hengst, Wouter Vening, Yonta van der Burgh, Ahmad Ghazal, Hamza Ibrahim, Mourad Niazi, Bilal Alkhaffaf, Mohammad Altarawni, Giovanni Carlo Cesana, Marco Anselmino, Matteo Uccelli, Stefano Olmi, Christine Stier, Tahsin Akmanlar, Thomas Sonnenberg, Uwe Schieferbein, Alejandro Marcolini, Diego Awruch, Marco Vicentin, Eduardo Lemos de Souza Bastos, Samuel Azenha Gregorio, Anmol Ahuja, Tarun Mittal, Roel Bolckmans, Tom Wiggins, Clément Baratte, Judith Aron Wisnewsky, Laurent Genser, Lynn Chong, Lillian Taylor, Salena Ward, Lynn Chong, Lillian Taylor, Michael W Hi, Helen Heneghan, Naomi Fearon, Andreas Plamper, Karl Rheinwalt, Helen Heneghan, Justin Geoghegan, Kin Cheung Ng, Naomi Fearon, Krzysztof Kaseja, Maciej Kotowski, Tarig A Samarkandy, Adolfo Leyva-Alvizo, Lourdes Corzo-Culebro, Cunchuan Wang, Wah Yang, Zhiyong Dong, Manel Riera, Rajesh Jain, Hosam Hamed, Mohammed Said, Katia Zarzar, Manuel Garcia, Ahmet Gökhan Türkçapar, Ozan Şen, Edoardo Baldini, Luigi Conti, Cacio Wietzycoski, Eduardo Lopes, Tadeja Pintar, Jure Salobir, Cengiz Aydin, Semra Demirli Atici, Anıl Ergin, Huseyin Ciyiltepe, Mehmet Abdussamet Bozkurt, Mehmet Celal Kizilkaya, Nezihe Berrin Dodur Onalan, Mariana Nabila Binti Ahmad Zuber, Wei Jin Wong, Amador Garcia, Laura Vidal, Marc Beisani, Jorge Pasquier, Ramon Vilallonga, Sharad Sharma, Chetan Parmar, Lyndcie Lee, Pratik Sufi, Hüseyin Sinan, Mehmet Saydam

**Affiliations:** 1 Upper GI unit, University Hospital Birmingham NHS Foundation Trust, Birmingham, UK; 2 Institute of Metabolism and Systems Research (IMSR), College of Medical and Dental Sciences, University of Birmingham, Birmingham, UK; 3 Institute of Applied Health Research, Murray Learning Centre, University of Birmingham, Birmingham, UK; 4 Institute of Cancer and Genomic Sciences, University of Birmingham, Birmingham, UK; 5 NIHR Biomedical Research Centre, Birmingham, B15 2TT UK; 6 NIHR Surgical Reconstruction and Microbiology Research Centre, Birmingham, B15 2TT UK; 7 MRC Health Data Research UK (HDR), Midlands Site, UK; 8 Centre for Endocrinology, Diabetes and Metabolism (CEDAM), Birmingham Health Partners, Birmingham, UK; 9 Department of Diabetes and Endocrinology, University Hospitals Birmingham NHS Foundation Trust, Birmingham, UK; 10 Bariatric Unit, South Tyneside and Sunderland NHS Trust, Sunderland, UK

**Keywords:** COVID-19, SARS-CoV-2, Pandemic, Bariatric surgery, Obesity surgery, Revisional surgery

## Abstract

**Background:**

There are data on the safety of cancer surgery and the efficacy of preventive strategies on the prevention of postoperative symptomatic COVID-19 in these patients. But there is little such data for any elective surgery. The main objectives of this study were to examine the safety of bariatric surgery (BS) during the coronavirus disease 2019 (COVID-19) pandemic and to determine the efficacy of perioperative COVID-19 protective strategies on postoperative symptomatic COVID-19 rates.

**Methods:**

We conducted an international cohort study to determine all-cause and COVID-19-specific 30-day morbidity and mortality of BS performed between 01/05/2020 and 31/10/2020.

**Results:**

Four hundred ninety-nine surgeons from 185 centres in 42 countries provided data on 7704 patients. Elective primary BS (n = 7084) was associated with a 30-day morbidity of 6.76% (n = 479) and a 30-day mortality of 0.14% (n = 10). Emergency BS, revisional BS, insulin-treated type 2 diabetes, and untreated obstructive sleep apnoea were associated with increased complications on multivariable analysis. Forty-three patients developed symptomatic COVID-19 postoperatively, with a higher risk in non-whites. Preoperative self-isolation, preoperative testing for SARS-CoV-2, and surgery in institutions not concurrently treating COVID-19 patients did not reduce the incidence of postoperative COVID-19. Postoperative symptomatic COVID-19 was more likely if the surgery was performed during a COVID-19 peak in that country.

**Conclusions:**

BS can be performed safely during the COVID-19 pandemic with appropriate perioperative protocols. There was no relationship between preoperative testing for COVID-19 and self-isolation with symptomatic postoperative COVID-19. The risk of postoperative COVID-19 risk was greater in non-whites or if BS was performed during a local peak.

**Supplementary Information:**

The online version contains supplementary material available at 10.1007/s11695-021-05493-9.

## Introduction

The coronavirus disease 2019 (COVID-19) pandemic has put a severe strain on global healthcare resources. This along with the realisation that perioperative severe acute respiratory syndrome coronavirus 2 (SARS-CoV-2) infection is associated with high 30-day mortality (23.8%) [1] led to the cancellation of millions of elective and semi-urgent surgical procedures worldwide, including bariatric and metabolic surgery (BS) [2].

It is also known that patients who inadvertently underwent BS before the scale of the pandemic became fully apparent and developed perioperative SARS-CoV-2 infection suffered significant morbidity [3]. There were hence genuine concerns that the morbidity and mortality of BS might be higher during the pandemic. This led to several consensus statements and guidelines on the safe resumption of BS [4–6]. The effect of adoption of these consensus statements on the morbidity and mortality of BS was, however, unknown. Knowledge of the factors that are associated with morbidity and postoperative COVID-19 infection might further allow us to improve our recommendations for safe BS during the pandemic.

Recommendations for safe surgery during the pandemic include routine preoperative reverse-transcriptase polymerase chain reaction (RT-PCR) testing for SARS-CoV-2 and/or preoperative self -isolation for variable time durations [7–11]. Studies from cancer surgery further suggest that a negative preoperative RT-PCR or operating in hospitals that have “coronavirus disease (COVID-19) free” surgical pathways (complete segregation of the operating theatre, critical care, and inpatient ward areas) is associated with a lower rate of postoperative pulmonary complications [12, 13]. However, data regarding the safety of non-cancer surgery such as BS during the COVID-19 pandemic and whether the above-mentioned pathways result in safer BS outcomes is unknown.

Associations between obesity and severe COVID-19 [14, 15]; patients’ concerns regarding the availability and safety of BS during the pandemic [16, 17]; and the negative impact of the pandemic and lockdowns on the health and eating and physical activity behaviours [18] further make it important to examine the safety of BS during the COVID-19 pandemic.

We, therefore, conducted an international cohort study — the global 30-day outcomes of bariatric surgEry iN thE coVid-19 erA (GENEVA) to capture 30-day morbidity and mortality of BS performed during the COVID-19 pandemic. The results of the first 2001 patients who had their BS between 1st May and 10th July 2020 were reported a few months ago [19]. However, since July 2020, there have been further peaks of COVID-19 globally. Hence, it was essential to continually monitor practice to ensure the safety of BS and explore predictors of complications and postoperative COVID-19.

As a result, we continued to collect data and expanded the study to include 5703 more patients’ data submitted by 281 more surgeons from 58 more centres in 7 more countries. The study hypothesis was that the 30-day morbidity and mortality of BS performed globally during the COVID-19 pandemic would be similar to the pre-pandemic figures. The study’s primary outcome measure was all-cause and COVID-19-specific 30-day morbidity and mortality of BS. Secondary outcome measures were predictors of BS complications during the pandemic and the effect of preoperative testing, preoperative self-isolation, hospital protocols, and local pandemic burden on postoperative COVID-19 rates.

## Methods

### Study Design and Population

We conducted a global, multicentre, observational cohort study of BS (elective primary, elective revisional, and emergency) in adults (≥ 18 years) performed between 1/05/2020 and 31/10/2020. The study start date was 1st May to exclude patients who underwent BS before the full scale of the pandemic and its effect on surgical patients became widely known.

We included all consecutive adult patients undergoing elective BS between 1st May and 31st October 2020 regardless of the surgical approach or the patient’s preoperative COVID-19 status. All laparoscopic, open, robotic, or hybrid surgical procedures were included. Laparoscopic sleeve gastrectomy, Roux-en-Y gastric bypass, and one anastomosis gastric bypass procedures were identified separately, whereas all others were pooled together in the “others” category. Procedures included in the “others” category were a range of diverse bariatric procedures. Data was also collected for patients undergoing emergency surgery related to previous bariatric surgery. The participating centres and surgeons were contacted using personal networks and national professional BS societies (via newsletters, email, and social media groups such WhatsApp®) and bariatric professionals’ networks on LinkedIn®, Facebook®, and Twitter®.

This project was registered as a multinational audit (number: 5197) at the University Hospitals Birmingham NHS Foundation Trust, UK. Each site project lead was responsible for obtaining local governance approvals and data sharing agreements before entering data into the registry. Patient’s approval to share their anonymised data was obtained by the individual collaborators and it was the responsibility of the site leads to ensure that patient approval was in place and documented in the notes before entering data into the registry. The site leads had to agree to these terms electronically before they were allowed access to the registry to enter data.

### Data Collected and Handling

Data collection included patients’ demographics, details of surgery performed, preoperative COVID-testing protocols and outcomes, in-hospital and 30-day COVID-19, and surgery-specific morbidity and mortality. If a patient developed more than one complication, additional questions had to be completed for each complication. This information was collected using 77 questions (supplementary file).

Complications were captured using the Clavien-Dindo (CD) classification system [20] for reporting surgical complications. This allowed for easier comparison of complication data and captured all complications irrespective of their severity. We further captured individual complications that would be important to the bariatric community such as bleeding and leak rates and complications such as chest infection/pneumonia that would be important in the context of the COVID-19 pandemic. In case of more than one complication occurring in the same patient, the highest CD score was reported.

Data collected regarding the centre and the surgeon were organised in 73 questions and included extensive profiling of the centre, the surgeon, and the impact and handling of COVID-19 in that centre. A copy of the questions can be found in the appendix.

Study data were collected and managed using REDCap electronic data capture tools hosted at the University of Birmingham, UK. REDCap (Research Electronic Data Capture) is a secure, web-based software platform designed to support data capture for research studies [21, 22].

Data entered on REDCap were examined weekly for any missing or erroneous data throughout the study period, and site leads were contacted for clarification. Collaborators were routinely contacted at 32 days following surgery if the 30-day follow-up data had not been completed. The final dataset was downloaded on the 10th of December once data queries had been resolved. Data was subsequently re-examined for omissions or abnormalities.

### Statistical Methods

Continuous data were presented as mean ± standard deviation (SD) or median (IQR) depending on data distribution. Frequencies were used to summarise categorical variables. Independent t-test or Mann-Whitney U test was used to examine differences between continuous variables depending on data distribution. A Chi-square test was used to compare categorical variables. Statistical analysis was performed using Statistical Package for the Social Sciences (SPSS) statistical software, version 27.0 (SPSS Inc).

The CD scale was used to assess outcomes in an ordinal logit regression model. Variable selection was parsimonious, with a small number of clinically determined predictors being selected pragmatically, guided by univariate analysis. This reduced the risk of overfitting or spurious associations. Variables were checked iteratively using Akaike information criterion scores to validate their inclusion in the multivariate model. Checks were made for non-linear associations and interactions were explored, though none were statistically significant so a main-effects only model was used. The effect sizes reported are odds ratios, specifically the odds of increasing from one complication level to a higher one. A link test was performed which gave a non-significant result suggesting that the model was not severely misspecified. StataCorp. 2015. (College Station, TX: StataCorp LP.) was used to produce the model. A p value of < 0.05 was considered significant unless stated otherwise.

To examine the relationship between the community burden of COVID-19 pandemic and symptomatic post-operative COVID-19, daily cumulative infection data were downloaded from John’s Hopkins University git repository [23] and differentiated to obtain daily numbers of new infection cases. To analytically define peak maxima of new infections, data curves for each country were fed through a low-pass Butterworth filter. Maxima were automatically detected if local maxima had a width of at least 7 days and reached at least 15% of the maximum number of infections of the country.

## Results

A total of 499 surgeons from 185 centres in 42 countries submitted data on 7734 patients who underwent BS between 1st May 2020 and 31st October 2020 at the participating centres. Of these, complete 30-day morbidity and mortality data were available for 7704 (99.6%) by the 10th of December 2020. Table 1 provides the basic demographics of these patients. Most patients (7084, 91.9%) underwent elective primary BS while 449 (5.8%) and 171 (2.2%) patients underwent elective revisional BS and emergency surgery following a previous BS respectively. The primary surgery population mainly consisted of young white females (5197; 73.4%) with severe obesity and a high prevalence of obesity comorbidities; a quarter of the study population were non-whites (1813; 25.59%).

**Table 1:** Basic demographic details of all patients undergoing elective primary BS, elective revisional BS, and emergency procedures

		Elective primary surgery					Elective revisional surgery			Emergency surgery
		All primary procedures	LSG	RYGB	OAGB	Others*	All revisional procedures	Conversional surgery	Other revisional procedures	
Total number		7084	3988	2091	705	300	449	285	164	171
Age		40.35 ± 11.9	38.78 ± 11.9	43.01 ± 11.4	41.13 ± 11.3	40.86 ± 11.9	44.81 ± 10.9	43.76 ± 10.9	46.64 ± 10.7	45.29 ± 11.9
Sex (M: F)		1886 (26.6%): 5197 (73.4%) 1 (0.01%)**	1100 (27.6%): 2888 (72.4%)	496 (23.7%): 1594 (76.2%) 1 (0.01%)**	208 (29.5%): 497 (70.5%)	82 (27.3%): 218 (72.7%)	84 (18.7%): 365 (81.3%)	56 (19.6%): 229 (80.4%)	28 (17.1%): 136 (82.9%)	27 (15.8%): 144 (84.2%)
Weight (in kg)		119.49 ± 24.4	119.51 ± 25.6	118.81 ± 21.2	122.46 ± 26.1	116.91 ± 25.2	104.35 ± 24.8	108.1 ± 24.1	97.85 ± 24.6	87.54 ± 24.8
BMI		43.03 ± 6.9	43.14 ± 7.4	42.52 ± 5.7	44.19 ± 7.5	42.29 ± 7.8	38.05 ± 8.8	39.51 ± 8.6	35.52 ± 8.6	31.08 ± 7.9
Ethnicity data										
White		5271 (74.41%)	3018 (75.68%)	1445 (69.11%)	541 (76.74%)	267 (89%)	374 (83.3%)	232 (81.4%)	142 (86.59%)	155 (90.64%)
Non-white***		1813 (25.59%)	970 (24.32%)	646 (31.89%)	164 (23.26%)	33 (11%)	75 (16.7%)	53 (18.6%)	22 (13.41%)	16 (9.36%)
Asian										
Black or African				108 (5.16%)		14 (4.67%)	19 (4.23%)	13 (4.56%)	6 (3.66%)	2 (1.17%)
American		398 (5.62%)	184 (4.61%)	34 (1.63%)	92 (13.05%)	2 (0.67%)	10 (2.23%)	4 (1.4%)	6 (3.66%)	6 (3.51%)
Hispanic or Latino		87 (1.23%)	51 (1.28%)	500 (23.91%)	0	17 (5.67%)	45 (10.02%)	35 (12.28%)	10 (6.1%)	7 (4.09%)
Native Hawaiian or other		1394 (18.41%)	716 (17.95%)	1 (0.05%)	71 (10.07%)	0	1 (0.22%)	1 (0.35%)	0	0
Pacific Islander		14 (0.2%)	12 (0.3%)	3 (0.14%)	1 (0.14%)	0	0	0	0	1 (0.58%)
American Indian or Alaska Native		10 (0.14%)	7 (0.18%)		0					
Co-morbidity data										
Any co-morbidity		4879 (68.9%)	2546 (63.86%)	1555 (74.37%)	564 (80.11%)	214 (71.33%)	260 (57.91%)	178 (62.46%)	82 (50%)	74 (43.27%)
Type 2 diabetes mellitus	Diet controlled	421 (5.9%)	205 (5.14%)	109 (5.21%)	76 (10.8%)	31 (10.33%)	18 (4.01%)	9 (3.16%)	9 (5.49%)	7 (4.09%)
	Oral medication	860 (12.1%)	392 (9.83%)	296 (14.16%)	113 (16.05%)	59 (19.67%)	34 (7.57%)	25 (8.77%)	9 (5.49%)	8 (4.68%)
	Insulin dependent	253 (3.6%)	75 (1.88%)	101 (4.83%)	49 (6.96%)	28 (9.33%)	14 (3.12%)	10 (3.51%)	4 (2.44%)	3 (1.75%)
Hypertension		2189 (30.9%)	1100 (27.59%)	724 (34.62%)	275 (39.06%)	90 (30%)	113 (25.17%)	82 (28.77%)	31 (18.9%)	37 (21.64%)
Sleep apnoea	Not on CPAP	857 (12.1%)	449 (11.26%)	221 (10.57%)	155 (22.02%)	32 (10.67%)	21 (4.68%)	19 (6.67%)	2 (1.22%)	13 (7.6%)
	on CPAP	949 (13.4%)	529 (13.27%)	297 (14.2%)	76 (10.8%)	47 (15.67%)	35 (7.8%)	25 (8.77%)	10 (6.1%)	3 (1.75%)
Hypercholesterolaemia		1523 (21.5%)	762 (19.11%)	476 (22.76%)	224 (31.82%)	61 (20.33%)	49 (10.91%)	38 (13.33%)	11 (6.71%)	22 (12.87%)
Others		2061 (29.1%)	1076 (26.99%)	656 (31.37%)	243 (34.52%)	86 (28.67%)	123 (27.39%)	86 (30.18%)	37 (22.56%)	35 (20.47%)
Smoking status										
Current smoker		1039 (14.7%)	647 (16.23%)	195 (9.33%)	113 (16.05%)	84 (28%)	67 (14.9%)	42 (14.74%)	25 (15.24%)	32 (18.71%)
Ex-smoker		928 (13.1%)	431 (10.81%)	388 (18.56%)	62 (8.81%)	47 (15.67%)	91 (20.3%)	56 (19.65%)	35 (21.34%)	35 (20.47%)
Non-smoker		5114 (72.2%)	2909 (72.96%)	1507 (72.07%)	529 (75.14%)	169 (56.33%)	291 (64.8%)	187 (65.61%)	104 (63.41%)	103 (60.23%)

*Other primary procedures: *SASI* single anastomosis sleeve ileal bypass, banded sleeve, *SADI-S* single anastomosis duodeno-ileal bypass with sleeve gastrectomy, open primary procedures, robotic primary procedures, resectional gastric bypass, gastroplication with OAGB, gastroplication, *SAGI* single anastomosis gastro-ileal bypass, *BPD* bilio-pancreatic diversion

**denotes transgender

***Non-white: Asian, Black or African American, Hispanic or Latino, Native Hawaiian or other Pacific Islander, and American Indian or Alaska Native

Of patients undergoing primary BS, laparoscopic sleeve gastrectomy (LSG), laparoscopic Roux-en-Y gastric bypass (LRYGB), and laparoscopic one anastomosis gastric bypass (LOAGB) were performed in 3988 (51.8%), 2091 (14.2%), and 705 (9.2%) patients, respectively. Three hundred (3.9%) patients underwent “others” procedures. Other procedures included a range of diverse bariatric surgical procedures and also procedures performed using open, robotic, and hybrid surgical approaches.

### 30-Day Morbidity and Mortality (Table 2)

Four hundred seventy-nine (6.76%) patients undergoing primary BS developed at least one complication. Of these, 302 (4.26%) were minor CD grade I or II; and 177 (2.5%) were CD grade III, IV, or V. Overall, 49 (0.69%) patients required management in intensive care (CD grades IV and V). Ten (0.14%) patients died. Although complication rates were higher in patients undergoing elective revisional surgery (n = 53/449, 11.8%) and those undergoing emergency surgery (n = 35/171, 20.46%) compared to primary BS, no deaths were reported for these groups.

**Table 2:** 30-day morbidity and mortality of elective primary BS, elective revisional BS, and emergency procedures

	Primary surgery					Revisional surgery			Emergency surgery
	All primary procedures	LSG	RYGB	OAGB	Others	All revisional procedures	Conversional surgery	Others	
	7084	3988	2091	705	300	449	285	164	171
Highest grade									
Clavien Dindo grade I	166 (2.34%)	84 (2.11%)	63 (3.01%)	11 (1.56%)	8 (2.67%)	15 (3.34%)	11 (3.86%)	4 (2.44%)	12 (7.02%)
Clavien Dindo grade II	136 (1.92%)	63 (1.58%)	48 (2.3%)	17 (2.41%)	8 (2.67%)	11 (2.45%)	6 (2.11%)	5 (3.05%)	11 (6.43%)
Clavien Dindo grade IIIa	33 (0.47%)	16 (0.4%)	8 (0.38%)	7 (0.99%)	2 (0.67%)	8 (1.78%)	5 (1.75%)	3 (1.83%)	1 (0.58%)
Clavien Dindo grade IIIb	95 (1.34%)	50 (1.25%)	31 (1.48%)	12 (1.7%)	2 (0.67%)	13 (2.9%)	8 (2.81%)	5 (3.05%)	10 (5.85%)
Clavien Dindo grade IVa	32 (0.45%)	13 (0.33%)	15 (0.72%)	3 (0.43%)	1 (0.33%)	5 (1.11%)	4 (1.4%)	1 (0.61%)	1 (0.58%)
Clavien Dindo grade IVb	7 (0.1%)	3 (0.08%)	2 (0.1%)	0	2 (0.67%)	1 (0.22%)	1 (0.35%)	0	0
Clavien Dindo grade V	10 (0.14%)	4 (0.1%)	0	4 (0.57%)	2 (0.67%)	0	0	0	0
All complications	479 (6.76%)	233 (5.84%)	167 (7.98%)	54 (7.65%)	25 (8.33%)	53 (11.8%)	35 (12.2%)	18 (10.98%)	35 (20.46%)
Clavien Dindo grade I and II	302 (4.26%)	147 (3.68%)	111 (5.30%)	28 (3.97%)	16 (5.33%)	26 (5.79%)	17 (5.96%)	9 (5.49%)	23 (13.45%)
Clavien Dindo grade III, IV, V	177 (2.50%)	86 (2.15%)	56 (2.67%)	26 (3.68%)	9 (3.0%)	27(6.01%)	18 (6.31%)	9 (5.49%)	12 (7.01%)
COVID infection									
COVID-19	38 (0.54%)	20 (0.5%)	10 (0.48%)	5 (0.71%)	3 (1%)	2 (0.45%)	0	2 (1.22%)	3 (1.75%)
Specific complications									
Bleeding	120 (1.69%)	57 (1.43%)	41 (1.96%)	20 (2.84%)	2 (0.67%)	9 (2%)	5 (1.75%)	4 (2.44%)	0
Leak from gastrointestinal tract	50 (0.71%)	26 (0.65%)	13 (0.62%)	9 (1.28%)	2 (0.67%)	9 (2%)	8 (2.81%)	1 (0.61%)	2 (1.17%)
Wound infection	42 (0.59%)	20 (0.5%)	11 (0.53%)	7 (0.99%)	4 (1.33%)	11 (2.45%)	4 (1.4%)	7 (4.27%)	6 (3.51%)
Postoperative pneumonia (not otherwise specified)	15 (0.21%)	5 (0.13%)	7 (0.33%)	1 (0.14%)	2 (0.67%)	3 (0.67%)	2 (0.7%)	1 (0.61%)	3 (1.75%)
DVT	3 (0.04%)	2 (0.05%)	1 (0.05%)	0	0	0	0	0	1 (0.58%)
PE	7 (0.1%)	2 (0.05%)	3 (0.14%)	1 (0.14%)	1 (0.33%)	0	0	0	0
Other*	244 (3.44%)	112 (2.81%)	93 (4.45%)	25 (3.55%)	14 (4.67%)	32 (7.13%)	22 (7.72%)	9 (5.49%)	27 (15.79%)

*Other complications: anaesthetic complications, atelectasis, bowel obstruction, cardiovascular changes, constipation, dehydration, dermatological changes, diarrhoea, dumping syndrome, dysphagia, electrolyte imbalance, fistula, hernia, hyperglycaemia, hypertension, hypoglycaemia, hypotension, ileus, inflammation of local structures, intra-abdominal abscess formation, intraoperative damage to local structures, myocardial infarction, nausea, pain, perforation, port-site haematoma, postoperative pyrexia, reflux, renal failure, type 1 and 2 respiratory failure, ulceration, urinary tract infection, venous thromboembolism (non DVT/PE), and wound dehiscence

Factors affecting complications have been described in Table 3. On logit regression analysis, increasing age, male sex, being a current or former smoker (vs. non-smoker), having insulin-dependent type 2 diabetes (T2D; vs. patients who did not have diabetes), obstructive sleep apnoea (OSA) not on continuous positive airways pressure (CPAP) (vs. patients did not have OSA), hypertension, and hypercholesterolaemia were associated with increased complication levels. Emergency procedures and revisional procedures were associated with higher odds of increased complications, as was primary LRYGB or other primary procedures as compared to primary LSG. Prior experience of the surgeon also appeared significant.

**Table 3:** Univariate and logit regression analysis (7687 observation) for the risk of postoperative surgical complications within 30 days. *** p < 0.01, ** p < 0.05, * p < 0.1

		Univariate analysis			Regression analysis	
		No complication (7137 patients)	Complication (567 patients)	p value	Odds ratio	Confidence intervals
Age (year increase)		40.52 ± 11.8	43.14 ± 12	< 0.001	1.008*	1.000–1.017
Sex = male		1822 (25.53%)	175 (30.86%)	0.02	1.259**	1.033–1.534
BMI (unit increase)		42.5 ± 7.3	41.9 ± 8.4	0.71	0.993	0.981–1.006
Non-white		1755 (24.9%)	129 (22.8%)	0.26	1.020	0.797–1.305
Type 2 diabetes mellitus	Diet controlled	398 (5.6%)	41 (7.2%)	< 0.001	1.141	0.800–1.630
	Oral medication	790 (11.1%)	54 (9.5%)		0.681**	0.497–0.931
	Insulin dependent	233 (3.3%)	37 (6.5%)		1.451*	0.987–2.134
Hypertension		1434 (20.1%)	161 (28.4%)	< 0.001	1.083	0.879–1.334
Sleep apnoea	Not on CPAP	802 (11.2%)	89 (15.7%)	0.001	1.107	0.842–1.457
	on CPAP	902 (12.6%)	85 (15%)	0.107	1.395**	1.076–1.809
Hypercholesterolemia		1434 (20.1%)	161 (28.4%)	< 0.001	1.440***	1.161–1.786
Other co-morbidities					1.062	0.874–1.290
Smoking status (past or current)		1988 (27.9%)	204 (36%)	< 0.001	1.253**	1.038–1.513
Surgery category reference — primary LSG		3755 (52.6%)	233 (41.1%)	< 0.001		
Surgery category	Emergency surgery	136 (1.91%)	35 (6.17%)		3.089***	1.983–4.814
	Revisional surgery	396 (5.5%)	53 (9.3%)		1.927***	1.374–2.701
	RYGB	1924 (27%)	167 (29.5%)		1.219*	0.978–1.521
	OAGB	651 (9.1%)	54 (9.5%)		1.244	0.902–1.716
	Other primary surgeries	275 (3.9%)	25 (4.4%)		1.492*	0.931–2.392
Hospital size reference < 200 beds		2127 (29.8%)_	134 (23.6%)	< 0.001		
Hospital capacity (beds)	200– 499	2156 (30.2%)	161 (28.4%)		1.068	0.818–1.395
	500–999	1886 (26.4%)	194 (34.2%)		1.140	0.841–1.545
	1000–1999	691 (9.7%)	64 (11.3%)		1.191	0.831– 1.707
	> 2000	277 (3.9%)	14 (2.5%)		0.735	0.383–1.409
Hospital category reference — district general		2247 (31.5%)	145 (25.6%)	< 0.001		
Hospital category	Teaching hospital	2254 (31.6%)	217 (38.3%)		1.431***	1.105–1.852
	University hospital	2636 (36.9%)	205 (36.2%)		1.088	0.836–1.414
Surgeon experience reference < 500 procedures		1129 (15.8%)	110 (19.4%)	< 0.001		
Surgeon experience (procedures)	500–999	827 (11.6%)	35 (6.2%)		1.167	0.862 - 1.581
	1000–2000	4264 (59.7%)	322 (56.8%)		0.754**	0.587–0.968
	> 2000	917 (12.8%)	100 (17.6%)		0.434***	0.285–0.659

Of the 10 patients who died [5 LSG, 4 LOAGB, and 1 laparoscopic single anastomosis duodeno-ileal bypass patients], seven (70.0%) were females. Four of these died due to leaks (3 following LOAGB and 1 following LSG), two died due to pulmonary embolism (PE), one died of COVID-19 pneumonia with PE, one died of mesenteric thrombosis, one died of bleeding, and one died of multi-organ failure (not otherwise specified; further data was not available for this patient).

The patient who was diagnosed with COVID pneumonia postoperatively had been advised to self-isolate for 2 weeks preoperatively and also had a negative RT-PCR preoperatively. Of the remaining 9 patients, 3 (33.3%) had been advised preoperative self-isolation; 6 (66.6%) had preoperative testing for SARS-CoV-2.

### Perioperative COVID-19 Protocols (Table 4)

Overall, 54% (n = 4068) of elective (primary and revisional) patients were not recommended any preoperative self-isolation. Similarly, 19.8% (n = 1491) of the patients did not undergo any preoperative testing to rule out SARS-CoV-2 infection. One hundred thirty-six out of 185 centres indicated that they were treating COVID-19 patients in the same hospitals (6086 patients; 79%), as opposed to 49 centres where the facility was not treating COVID-19 patients (1618 patients; 21%). Testing of staff was performed in 67 centres (2144 patients; 27.8%).

**Table 4:** Perioperative COVID-19 safety protocols

Was patient asked to self (or home) isolate before the surgery?						
	Elective surgery (n = 7533)			Emergency surgery (n = 171)		
Yes, for approximately 1 week	2067 (27.4%)			1 (0.6%)		
Yes, for approximately 10 days	163 (2.2%)			2 (2.1%)		
Yes, for approximately 2 weeks	1061 (14.1%)			22 (12.9%)		
No self-isolation recommended	4068 (54%)			119 (69.6%)		
Other durations	174 (2.3%)			27 (15.8%)		
Preoperative testing						
Did the patient undergo any specific test for SARS-CoV-2/ COVID-19 preoperatively to rule out active infection or confirm immunity?	6042 (80.2%)			93 (54.4%)		
Name of test	Positive	Negative	Not tested/not available	Positive	Negative	Not tested/ Not available
RT PCR	11 (0.15%)	5193 (68.94%)	838 (11.12%)	2 (1.17%)	72 (42.11%)	19 (11.11%)
Antigen test	0	279 (3.7%)	5757 (76.42%)	0	23 (13.45%)	70 (40.94%)
Antibody test	17 (0.23%)	671 (8.91%)	5344 (70.94%)	0	3 (1.75%)	90 (52.63%)
Chest X ray	9 (0.12%)	3593 (47.7%)	2424 (32.18%)	3 (1.75%)	38 (22.22%)	52 (30.41%)
CT scan chest	9 (0.12%)	1163 (15.44%)	4852 (64.41%)	1 (0.58%)	13 (7.6%)	79 (46.2%)
Was PPE (FFP3/N95) used in theatre?	Yes 3316 (44.02%) No 4217 (55.98%)			Yes 49 (28.65%) No 122 (71.35%)		

### Postoperative COVID-19

Of the 42 countries, 34 countries had at least one peak of COVID-19 during the study period (Table 5). A total of 6092 (79%) patients were entered into the database from these countries [23].

**Table 5:**
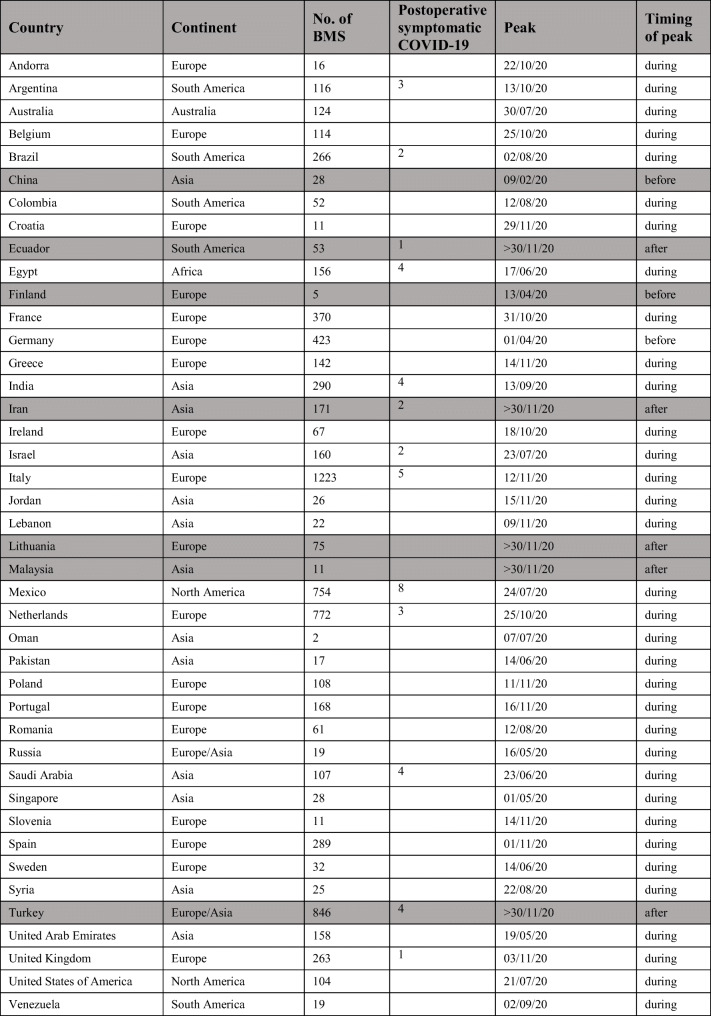
Country-wise reporting of cases and the relationship of the peak incidence of COVID-19 to the study period (1st May–30th Nov). *(Based on* *https://github.com/CSSEGISandData/COVID-19/tree/master/csse_covid_19_data/csse_covid_19_time_series**accessed 01/12/2020 at 14:00 GMT).* Shaded lines represent countries that did not have a peak of COVID-19 incidence during the study period

Forty-three patients (0.56%) developed symptomatic COVID-19 postoperatively. These patients were from Mexico (n = 8), Italy (n = 5), Egypt (n = 4), India (n = 4), Saudi Arabia (n = 4), Turkey (n = 4), Argentina (n = 3), the Netherlands (n = 3), Brazil (n = 2), Iran (n = 2), Israel (n = 2), Ecuador (n = 1), and UK (n = 1). Thirty-eight had undergone elective primary BS; two had undergone elective revisional BS, and three had undergone emergency surgery. Further attributes for patients who developed symptomatic COVID-19 postoperatively are described in Table 6.

**Table 6:** Factors associated with symptomatic postoperative COVID-19

	Symptomatic postoperative COVID-19		p value
	Yes	No	
Non-white ethnicity	18 (41.8%)	1886 (24.6%)	0.013
Preoperative self-isolation	26 (60.5%)	3290 (44.1%)	0.031
Preoperative testing	35 (81.4%)	6100 (79.6%)	0.774
Hospitals also treating COVID-19 patients	32 (74.4%)	6054 (79%)	0.46
Use of PPE in theatre	23 (0.7%)	4319 (99.5%)	0.193

Majority of these were either CD grade I (n = 29; 67.4%) or grade II (n = 11; 25.6%) complications. There were two CD grade IV (4.65%) and one CD grade V (2.32%) complications. Thus, there was one mortality due to COVID-19 in this study. This patent was a 35-year-old female, who had a BMI of 70.0 kg/m^2^ along with, T2D on insulin, hypertension, OSA, hypercholesterolaemia, and previous history of deep vein thrombosis and underwent LOAGB. This patient had tested negative on RT-PCR and was recommended self-isolation for 2 weeks before surgery. She was discharged home the day after surgery and presented 10-days later with COVID-19 pneumonia. The initial surgery for this patient had been performed in a small-sized district general hospital which also treated COVID-19 patients concurrently. This patient had surgery during the top tertile of SARS-CoV-2 infection incidence for the country.

### Factors Associated with Symptomatic Postoperative COVID-19 (Table 6)

On univariate analysis of biological plausible variables, only non-white ethnicity was significantly associated with symptomatic post-operative COVID-19 (p = 0.013; white, 25/5800; 58.1% of COVID-19-positive patients; non-white, 18/1904; 41.9% of COVID-19-positive patients).

The proportion of people who were advised to self-isolate was greater in the cohort that developed COVID-19 compared to those who did not develop COVID-19 (60.5%; 26/43 vs. 44.1% 3290/7460, p = 0.03).

There was no significant difference in the proportion of patients who had preoperative testing for SARS-CoV-2 between those who did and did not develop postoperative symptomatic COVID-19 (81.4%; 35/43 vs 79.6%; 6100/7661).

Similarly, there was no difference in the proportion of patients who underwent BS in hospitals also looking after COVID-19 patients as compared to those who weren’t (74.4%; 32/43 vs 79.0%; 6054/7661).

### The Temporal Relationship Between the Country Incidence of COVID-19 and the Reported Post-BS Symptomatic COVID-19 Cases (Fig. 1)

With regard to the incidence of COVID-19 for individual countries, data from Ecuador, Iran, and Turkey were excluded from this analysis due to the absence of an obvious peak during the study period.

**Fig. 1:**
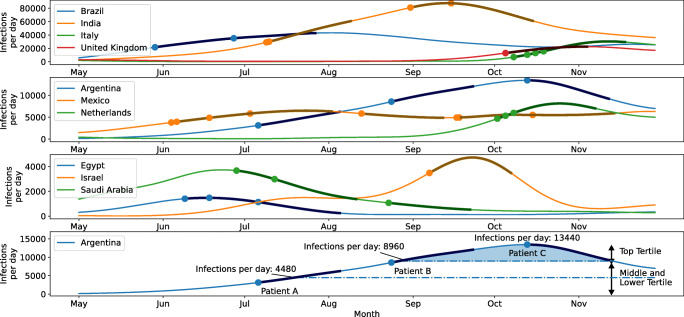
The temporal relationship between the country incidence of COVID-19 and the reported post-BS symptomatic COVID-19 cases. Daily cumulative infection data were downloaded from John’s Hopkins University git repository^19^ and differentiated to obtain daily numbers of new infection cases. In order to analytically define peak maxima of new infections, data curves for each country were fed through a low-pass Butterworth filter. Maxima were automatically detected if local maxima had a width of at least 7 days and reached at least 15% of the maximum number of infections of the country. Day of surgery is indicated for each SARS-CoV2 positive patient with a dot. The darker shaded area after each dot indicates the 30-day follow-up period. The study period started at 01/05/2020 and ended at 30/11/2020. The top three panels were used to show this data due to the differences in the scale of incidence of SARS-CoV-2 infection in respective countries. The bottom panel illustrates the tertiles and infections/ day in Argentina. Patient A had date of surgery and 30-day follow-up within the lower and middle tertile. Patient B had the 30-day follow-up in the top tertile and Patient C had date of surgery and 30-day follow-up in the top tertile of the number of cases

Symptomatic postoperative COVID-19 was more likely to develop (0.7%; 34/4674 cases) when all/part of the postoperative 30-day follow-up was within the top tertile of SARS-CoV-2 infection incidence for that country as opposed to when the incidence was in the bottom two tertiles (0.1%; 2/1924 cases). This difference was statistically significant (p < 0.001).

### Hospital and Surgeon Characteristics

Data was equally represented from district general hospitals vs. teaching hospitals vs. university hospitals (2392 (31%) vs. 2471 (32.1%) vs. 2841 (36.9%) patients). The majority of the data were from hospitals with a bed capacity of < 1000 beds–5152 (66.9%) patients. A majority of the surgeons had performed between 1000 and 2000 procedures — 4586 (59.5%).

## Discussion

This is the biggest study on patients undergoing any non-cancer surgery during the pandemic. This study showed that elective BS performed during the COVID-19 pandemic is probably safe with low 30-day morbidity and mortality and very low risk of symptomatic COVID-19, most of which were mild severity infections. This study also identified the factors associated with 30-day complications and symptomatic COVID-19 post-BS. These findings should reassure patients and health care providers regarding the safety of BS during the COVID-19 pandemic.

The burden of the pandemic has been uneven across the globe and also fluctuated widely from month to month within regions. At times, the healthcare systems have been too overwhelmed to allow for any elective surgery to take place. However, as is evident from our study, there have also been times when some surgical teams have had a more favourable pandemic condition and the resources to carry out elective surgery such as BS. This is probably a result of the massive global effort on the development of consensus guidelines and prioritisation criteria on safe resumption of BS [4, 6, 24]. But there is little data on the safety of the BS during the pandemic with the adoption of some or all of these recommendations. In particular, there is a need to understand the efficacy of protocols in identifying asymptomatic SARS-CoV-2 infected patients and reducing viral transmission within healthcare facilities. We also need to understand if other changes imposed by the pandemic such as recommendations to avoid laparoscopic surgery without any definite evidence [25]; deskilling of the surgical teams; recommendations to shorten hospital stay of patients and increased use of telemedicine[4, 6]; and recommendations to operate on highest risk patients first [24] have impacted the safety of BS.

In our first report from this dataset [19], we had concluded that the 30-day morbidity and mortality of elective primary BS at 6.8% and 0.05%, respectively, seemed similar to the pre-pandemic figures. These figures have remained broadly unchanged at 6.76% and 0.14% even though we now have 7084 elective primary BS patients as opposed to 2001 in our initial report [19]. These figures appear similar to large pre-pandemic BS datasets [26–28].

It would be particularly interesting to know what percentage of patients undergoing BS during the pandemic develops symptomatic COVID-19 postoperatively and the outcomes of those who were inadvertently operated on whilst positive for SARS-CoV-2 at the time of the surgery. This makes the emergency cohort in our study of particular interest especially because we know that patients with perioperative SARS-CoV-2 have significantly higher morbidity and mortality [1]. At the same time, the outcomes of the different cohorts — primary elective BS, primary revisional BS, and emergency surgery groups — have been clearly and separately presented in this study as they have different characteristics. Of note, two emergency patients in this study were positive for SARS-CoV-2 at the time of the surgery — one developed COVID pneumonia and the other remained symptomatic. Neither of the two died.

The incidence of symptomatic COVID-19 postoperatively was low in our study, and most of these were mild (CD grade I or II). This may be due to several factors such as low disease burden in the community or effective preoperative strategies or measures to reduce in-hospital transmission of the virus. Overall, it does suggest that measures adopted locally by bariatric surgery teams were effective in preventing postoperative COVID-19. The single COVID-related mortality reported was a high-risk patient with severe obesity, multiple co-morbidities who had also tested negative for SARS-CoV-2 infection preoperatively. This patient presented 10 days after surgery. Since the median incubation period of COVID-19 is around 4 days [29], this patient probably acquired the virus postoperatively. This might suggest a need to develop protocols for postoperative protection for high-risk patients. Such an approach would be supported by another study on benign surgery (Trauma and Orthopaedics) by Karayiannis and colleagues [30] where authors recommended the development of effective strategies to reduce “post-operative exposure to the virus”. However, specific lessons that were learnt by the team where this unfortunate event happened were not captured in this study. This needs to be examined in future studies on this topic. Similarly, based on just one mortality, it would be unreasonable to conclude that patients with significant co-morbidities should not be offered BS during the pandemic.

Blood coagulation disorders continue to be an area of much interest during this pandemic [31, 32]. Patients in this study who died of pulmonary embolism or mesenteric venous thrombosis were not positive for SARS-CoV-2. We could not obtain detailed information on the patient who died of multiorgan failure (NOS) as the patient was treated in a different facility and the original surgeon could not provide us with all the details. However, the surgeon did not report this patient as having suffered from postoperative COVID-19.

With regard to perioperative strategies, there is data on preoperative RT-PCR testing and COVID-free pathways in the context of cancer surgery [12, 13] but not for patients undergoing benign surgery. In this study, preoperative viral testing or surgeries in a facility that was not treating COVID-19 patients were not associated with less postoperative symptomatic COVID-19 infection. Interestingly, we found that those who were advised preoperative self-isolation were significantly more likely to develop postoperative symptomatic COVID-19. This could be due to an overall higher viral burden in those communities at the time of preoperative consultation/counselling. One could hypothesise that surgical teams in areas with higher viral community load were more likely to recommend preoperative self-isolation. It is a counterintuitive association that needs examination in further focussed studies. Furthermore, the true protective effect of preoperative self-isolation may have been masked by the preoperative testing for SARS-CoV-2 infection as the majority of the patients in this study underwent some form of testing.

The risk of developing postoperative symptomatic COVID-19 was greater when the surgery or part of the 30-day follow-up occurred during the peak of COVID-19 incidence for that country. This might suggest a need for measures to reduce postoperative viral exposure. However, this information cannot be used to determine the level of pandemic burden when it would be safe to perform BS in any particular region since this information can only be worked out retrospectively with our methodology (BS was safer when the country incidence was in the lower two tertiles as opposed to the higher tertile).

The data from this study supports data from previous studies that patients of non-white ethnicity were at a higher risk of symptomatic COVID-19 postoperatively [33]. This may be due to several factors as non-white patients in this study come from countries with diverse healthcare systems and disease burdens [34]. Even within the same country, non-white populations may be at higher risk due to socio-economic and cultural factors. In light of these findings, additional and more stringent COVID-19-specific perioperative protocols could be considered for non-white patients who are undergoing BS. These could include stricter preoperative testing and self-isolation; more stringent precautions such as mask-wearing; segregation from emergency patients; use of PPE in the healthcare facility; easy access to surgical teams postoperative; and postoperative self-isolation.

This is the first multinational study to examine complications of BS and the predictors of these complications. In this study, we found that increasing age, male sex, or current or former smoking predicted the severity of complications following BS. This has previously been documented in the literature [35]. Emergency and revisional bariatric surgery were associated with a higher risk of postoperative complications as was primary RYGB. This reflects the complexity of emergency and revisional BS. Similarly, a higher risk of complications with RYGB has been previously reported [36]. It is worth noting here that though complication rates were higher for both LRYGB and LOAGB (compared to LSG), this difference was only significant for LRYGB after adjustment for risk factors. This data also confirmed a higher risk of complications with insulin-treated T2D, untreated obstructive sleep apnoea, hypertension, and hypercholesterolaemia. The association of T2D with increased morbidity and mortality after BS has been previously reported [36]. The data supported that increasing surgical experience was associated with a lower risk of postoperative complications and that type of hospital affected the risk of postoperative complications.

The findings of this study might also be relevant to the wider category of minor and intermediate-risk elective surgical procedures in the context of the pandemic [37]. Until conclusive research on this subject, the findings of this study might suggest a role for postoperative isolation and additional protocols in non-white patients.

### Study Limitations

This study has several limitations. It only includes data of participating centres and may therefore not represent the real global picture. The outcomes of this study are only applicable to the study population and cannot be extrapolated to populations whose composition is different from the current study in terms of age, sex, and racial characteristics. Also, data reported from countries was not proportional to the prevalence of pre-pandemic bariatric surgery in those countries. Notably, the numbers from the USA and Brazil are fewer than expected. This may be due to several factors such as the pandemic burden in those countries at the time of the study or engagement of surgical teams within those countries with this study.

There was no data available for 30 patients. However, complete 30-day follow-up data on 99.6% of the patients should be considered satisfactory. Also, though all reasonable care was undertaken to ensure our collaborators knew the importance of submitting all consecutive patients during the study period, we cannot be certain of that. At the same time, repeated reminders on the importance of this should have encouraged it. Although data on self-isolation has been presented, no attempts were made to capture adherence to self-isolation or mode of self-isolation (co-self-isolation with household).

Only patients with symptomatic infection were reported by the collaborators. The local prevalence data and incidence of postoperative SARS-CoV-2 infection have been presented over the entire study period and have not been divided into monthly periods. Finally, data on complications were self-reported by surgical teams and may therefore not represent an accurate picture. There remains the possibility that complications were underreported by collaborators. Authors though hope that anonymous data collection would have reduced any underreporting of complications. Furthermore, no attempt was made by the authors to identify data from individual participants in this global collaborative study. Data were only analysed as a whole. We further encouraged reporting of complications by asking collaborators to actively confirm the absence of complications at the 30-day mark.

### Study Strengths

The study has several strengths such as a large sample size, the global reach of the study, and extensive data profiling. Almost 80% of the data was collected from countries that had at least one peak of COVID-19 during the study period. With 99.6%, 30-day follow-up data, authors have high confidence that these findings represent true figures in the participating centres. The use of a validated CD system of reporting complications for such a large dataset further adds to the robustness of our study. The large sample size allowed the examination of predictors of complications and postoperative symptomatic COVID-19.

## Conclusion

Bariatric and metabolic surgery can be performed safely during the COVID-19 pandemic with appropriate perioperative protocols. Non-white ethnicity and having surgery during a local peak of COVID-19 for that country were associated with a greater risk of symptomatic COVID-19 postoperatively. There was no relationship between preoperative testing for COVID-19/ preoperative self-isolation and incidence of symptomatic postoperative COVID-19 perhaps suggesting that the measures to reduce the postoperative viral exposure are equally important.

## Supplementary Information

ESM 1 (DOCX 1265 kb)
