# Supplementary material for: 30-Day Morbidity and Mortality of Bariatric Surgery During the COVID-19 Pandemic: a Multinational Cohort Study of 7704 Patients from 42 Countries
Source: Obes Surg. 2021 Jul 30;31(10):4272–88. doi: 10.1007/s11695-021-05493-9 (PMC8323543; doi:10.1007/s11695-021-05493-9)
Supplement: Supplementary file 1 — (DOCX 1265 kb) [file 11695_2021_5493_MOESM1_ESM.docx]

**Appendix: Table of Contents**

| **CATEGORY** | **PAGE NUMBER** |
| --- | --- |
| Data Collection Questionnaires | 2 - 23 |
